# Multiple Geminated Supernumerary Premolars: A Rare Case Report

**DOI:** 10.1155/2015/726458

**Published:** 2015-05-20

**Authors:** Atul Soin, Gaurav Sharma, Gayatri Soin, Anudeep Raina, Puneet Mutneja, Archna Nagpal

**Affiliations:** ^1^Department of Prosthodontics, Kalka Dental College, Meerut 250006, India; ^2^Department of Oral Medicine and Radiology, Sudha Rustagi College of Dental Sciences and Research, Faridabad, Haryana 121002, India; ^3^Department of Endodontics, Kalka Dental College, Meerut 250006, India; ^4^Department of Oral Medicine and Radiology, Saraswati Dhanwantari Dental College and Hospital and Postgraduate Research Institute, Parbhani, Maharashtra 431407, India; ^5^Department of Prosthodontics and Crown and Bridge, Saraswati Dhanwantari Dental College and Hospital and Postgraduate Research Institute, Parbhani, Maharashtra 431407, India; ^6^Department of Oral Medicine and Radiology, P.D.M. Dental College and Research Institute, Bahadurgarh, Haryana 124507, India

## Abstract

Supernumerary teeth may be defined as any teeth or tooth substance in excess of the usual configuration of 20 deciduous and 32 permanent teeth. Gemination is defined as an attempt by a single tooth bud to divide, with a resultant formation of either a large tooth with a bifid crown or two completely divided teeth throughout the crown and root. Geminated supernumerary premolar is a rarity and the possibility of multiple occurrences is even rarer. An exhaustive review of English literature and a PubMed search conducted using the terms “gemination” and “multiple geminated supernumerary” revealed no case of multiple geminated supernumerary premolars. We report a case of multiple geminated supernumerary premolars in a 23-year-old male.

## 1. Introduction

Supernumerary teeth are odontostomatologic anomaly characterized by the existence of excessive number of teeth in relation to the normal dental formula [1]. Supernumerary teeth may be unilateral or bilateral, single or multiple, and in maxilla or in mandible. Supernumerary premolars occur with a prevalence of 0.29–0.64% [2]. The occurrence of multiple (more than two) supernumerary teeth without any associated systemic conditions or syndromes, however, is a rare phenomenon and occurs in less than 1% of cases [3]. The presence of one or more supernumerary teeth in the dentition has been referred to as “hyperdontia” [1].

Gemination is a developmental disturbance of the shape of teeth and is usually recognized as a partial cleavage of a single tooth germ resulting in one root and one pulp space with two partially or totally separated crowns [4]. Though it occurs in both dentitions, it has a higher prevalence in deciduous teeth, with a higher frequency in anterior maxillary region. It is also observed with an equal gender predilection. Unilateral gemination has a prevalence rate of 0.5% and 0.1% in deciduous and permanent dentition, respectively, whereas bilateral occurrence is seen in 0.01% to 0.04% in primary dentition and in 0.02% to 0.05% in permanent dentition [5].

We report here an extremely rare case report of nonsyndromic multiple geminated supernumerary teeth which, to the best of our knowledge, is the first ever case reported in English literature.

## 2. Case Report

**Figure 1:**
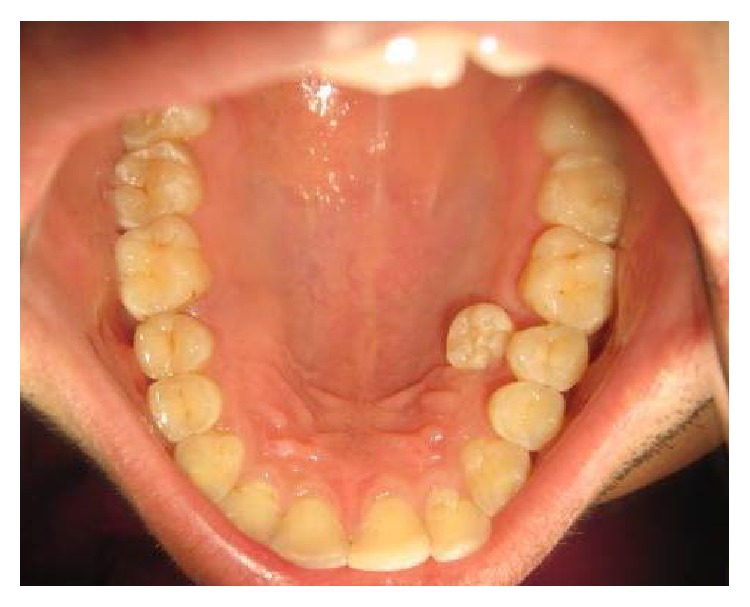
An intraoral clinical photograph (rotated) depicting a supernumerary premolar located palatally to maxillary premolars.

**Figure 2:**
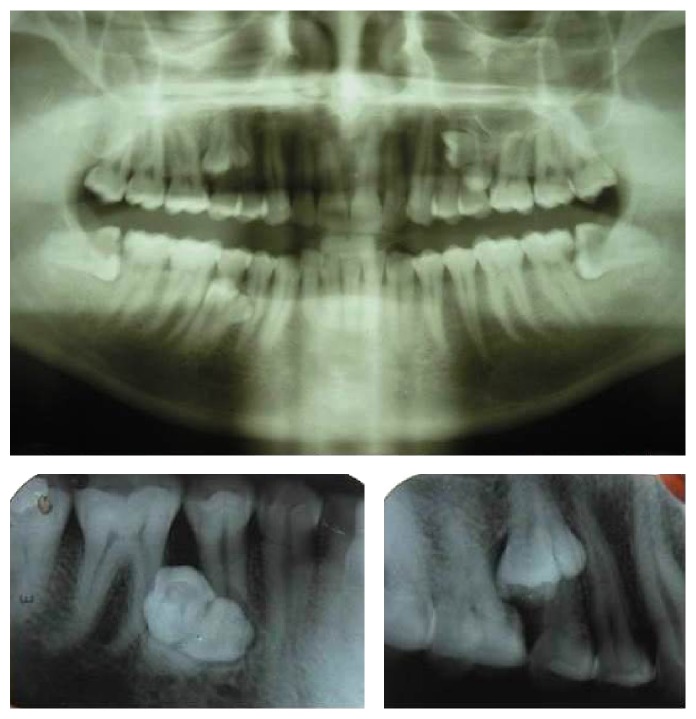
Panoramic radiograph showing the presence of impacted geminated premolars in left maxillary posterior region and right mandibular posterior quadrant. An intraoral periapical radiograph confirms the presence of geminated premolars in the respective regions.

**Figure 3:**
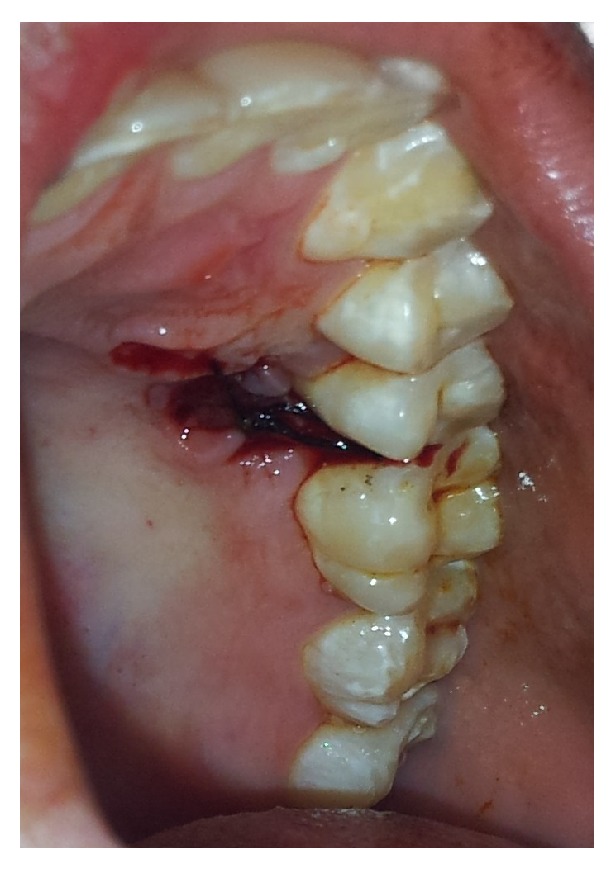
Intraoral clinical photograph depicting postextraction socket of the erupted maxillary supernumerary premolar.

A 23-year-old male presented to the department of oral medicine and radiology with chief complaint of extra tooth in upper left posterior region of jaw. There was no significant medical or family history. An intraoral clinical examination revealed supernumerary premolar present palatally to maxillary right premolars in first quadrant (Figure 1). There was no pain, swelling or mobility, or supernumerary tooth. In the mandibular right quadrant a bulge on the palatal aspect of mandibular premolars was also evident. The patient was advised of intraoral periapical radiographs that revealed the presence of geminated supernumerary premolars in the maxillary and mandibular right premolars area (Figure 2). Patient was advised of panoramic radiograph that further revealed the presence of geminated supernumerary premolar in left maxillary quadrant. Thus the patient was diagnosed with multiple, geminated, supernumerary premolars. The patient was advised of Cone beam computed tomography but, due to financial constraints, the patient was not willing for the same. The patient was subsequently advised of removal of supernumerary premolars. However the patient was willing only for erupted supernumerary premolar that was subsequently extracted under local anaesthesia with written informed consent from the patient (Figure 3). The patient was placed on follow-up.

## 3. Discussion

Supernumerary teeth may be defined as any teeth or tooth substance in excess of the usual configuration of 20 deciduous and 32 permanent teeth [1]. Gemination is defined as an attempt by a single tooth bud to divide, with a resultant formation of either a large tooth with a bifid crown or two completely divided teeth throughout the crown and root. In fusion there is a single crown with typically two roots or a single root with two canals. The frequency of gemination or fusion is 0.05% in the permanent dentition and the bilateral presentation is rare [6].

Multiple supernumerary teeth are frequently associated with a number of syndromes, namely, cleidocranial dysplasia, Gardner's syndrome, Fabry-Anderson syndrome, and cleft lip and palate [1]. Our patient had none of the above characteristics of the above conditions. The possibility of fused supernumerary premolars in the present case report was unlikely as there was an obvious bifid crown along with a single root in all three observed supernumerary premolars. The occurrence of gemination in a supernumerary tooth is very rare as only three cases of gemination in supernumerary premolars have been documented (Table 1) [4, 7, 8]. The present case is extremely rare and interesting as there was gemination of multiple supernumerary premolars in a nonsyndromic patient. An exhaustive review of English literature and a PubMed search conducted using the terms “gemination” and “multiple geminated supernumerary teeth” revealed no case of multiple geminated supernumerary premolars. Liu et al. had also proposed a new terminology of “geminated-premolar-like” for a similar condition [7]. All the three documented cases of solitary geminated supernumerary premolar and the current case have afflicted males indicating a possible gender predilection. The above finding can be coincidental as multiple supernumerary teeth tend to occur in males though gemination has no gender predilection [4, 7, 8].

The aetiology of gemination is still unknown though a complex interaction among a variety of environmental and genetic factors has been postulated. Geminated supernumerary premolar is a rarity and the possibility of multiple occurrences is even rarer. To the best of the authors' knowledge, there is no syndrome or condition associated with multiple geminated teeth (either anterior or premolars). A geminated tooth in anterior region may blemish aesthetics but the groove can also cause caries and a high plaque accumulation [5]. An impacted supernumerary tooth can also lead to complications like resorption of adjacent teeth, cystic spaces, and delayed eruption [4]. Hence, a geminated supernumerary tooth is more likely to cause complications due to its atypical anatomy. The authors opine that the impacted geminated supernumerary premolars should be removed as compared to supernumerary teeth that can still be kept on radiographic follow-up. Clinicians should be aware of this perplexing and rare entity that often looks subtle on appearance but the prognosis is different as compared to a normal tooth. An early diagnosis of impacted geminated supernumerary premolar can prevent the onset of possible complications and panoramic radiograph must be conducted if there is a presence of geminated supernumerary premolar.

## Figures and Tables

**Table 1:** A summary of reported cases of geminated supernumerary premolars.

Case	Author	Gender/age	Location
1	Liu et al. (2007) [7]	M/19	Mandibular premolar
2	Yang (2012) [8]	M/35	Maxillary premolar
3	Ather et al. (2012) [4]	M/19	Maxillary premolar

4	Present authors	M/23	Maxillary premolars and mandibular premolar
